# Brazilian Psychiatric Reform: Evolution and Challenges (2001-2024)

**DOI:** 10.1192/j.eurpsy.2025.1652

**Published:** 2025-08-26

**Authors:** M. A. Rodrigues

**Affiliations:** 1 Universidade Anhembi Morumbi, São Paulo, Brazil

## Abstract

**Introduction:**

The Brazilian psychiatric reform, initiated by Law 10.216 in 2001, brought a significant shift in the treatment of individuals with mental disorders. Focusing on deinstitutionalization and psychosocial care, the reform aimed to replace the hospital-centered model with a community-based services network. Over the past 23 years, this transformation has faced challenges, including the implementation of public policies, the guarantee of human rights, and the effective integration of mental health services.

**Objectives:**

To analyze the advances, challenges, and impacts of the Brazilian psychiatric reform from 2001 to 2024, with a focus on deinstitutionalization and psychosocial care.

**Methods:**

A documentary and bibliographic review was conducted, including legislation, government reports, scientific articles, and data from the Unified Health System (SUS). The research covered publications from 2001 to 2024 in the PubMed, Scielo, and Lilacs databases. The search terms used were “Brazilian psychiatric reform,” “deinstitutionalization,” and “psychosocial care.” The qualitative analysis highlighted legislative changes, service implementation, and challenges.

**Results:**

The psychiatric reform led to significant advances in the structuring of the Psychosocial Care Network (RAPS), including Psychosocial Care Centers (CAPS) and therapeutic residential services. There was a substantial reduction in psychiatric hospital beds and an expansion of community-based services. However, challenges persist, such as regional disparities in service provision and a shortage of qualified professionals.

The implementation of RAPS varied between regions, with the Southeast and South being more developed compared to the North and Northeast. Deinstitutionalization brought benefits, such as social reintegration of patients, but also revealed gaps, including the lack of support for families and strategies for managing crisis situations.

There is an ongoing need for public policies to support the reform, including adequate funding and a focus on human rights. Recent debates on the reintroduction of psychiatric hospitals indicate tensions in mental health policy, underscoring the need to reinforce the principles of the reform.

**Conclusions:**

From 2001 to 2024, the Brazilian psychiatric reform made progress in mental health care, emphasizing deinstitutionalization and the creation of a community-based network. However, challenges such as unequal service provision, a shortage of professionals, and political tensions indicate the need to strengthen the reform’s principles. Public policies should ensure humanized, integrated care based on the rights of individuals with mental disorders, ensuring the expansion of RAPS across the country.

**Disclosure of Interest:**

None Declared

